# New bounds for the exponential function with cotangent

**DOI:** 10.1186/s13660-018-1697-8

**Published:** 2018-05-08

**Authors:** Ling Zhu

**Affiliations:** Department of Mathematics, Zhejiang Gongshang University, Hangzhou, China

**Keywords:** 33B10, 26D05, Bounds, Inequalities, Circular functions

## Abstract

In this paper, new bounds for the exponential function with cotangent are found by using the recurrence relation between coefficients in the expansion of power series of the function $\ln (1-2x^{2}/15-px^{6})$ and a new criterion for the monotonicity of the quotient of two power series.

## Introduction

In 1978, Becker and Stark [1] proved the double inequality

$$\frac{8}{\pi^{2}-4x^{2}}< \frac{\tan x}{x}< \frac{\pi^{2}}{\pi^{2}-4x ^{2}} $$

or

$$-\frac{4x^{2}}{\pi^{2}}< x\cot x-1< \frac{ ( \pi^{2}-8 ) -4x ^{2}}{8} $$

holds for all $x\in (0,\pi /2)$. Since then, many inequalities for cotangent function were established by different ideas and methods; see e.g. [2–18]. Very recently, Lv, Yang, Luo, and Zheng [19] gave a new type of bounds for the function $\exp (x\cot x-1)$. More precisely, they proved the following results.

### Theorem A

*Let* $p,q\in ( -\infty,4/\pi^{2} ] $, $p^{\ast }\approx 0.13484$ *be the unique zero of the function* $\alpha_{p} ( \pi /2 ) -1$ *on* $( -\infty,4/\pi^{2} ) $, *where* $\alpha_{p} ( x ) =\exp ( x\cot x-1 ) / ( 1-px^{2} ) ^{1/ ( 3p ) }$ *if* $p\neq 0$ *and* $\alpha_{0} ( x ) =\exp ( x\cot x-1+x^{2}/3 ) $. *Then the double inequality*

1.1 $$\begin{aligned} \bigl( 1-px^{2} \bigr) ^{{1/ ( 3p ) }} < &e^{x\cot x-1} \\ < & \bigl( 1-qx^{2} \bigr) ^{{1/ ( 3q ) }} \end{aligned}$$

*holds for all* $x\in ( 0,\pi /2 ) $ *if and only if* $p\geq p^{\ast }$ *and* $q\leq 2/15\approx 0.13333$.

### Theorem B

*For* $x\in (0,\pi /2)$, *the double inequality*

1.2 $$ \biggl( 1-\frac{4}{3\pi^{2}}x^{2} \biggr) ^{\pi^{2}/4}< e^{x\cot x-1}< \biggl( 1-\frac{2}{15}x^{2} \biggr) ^{5/2} $$

*holds*.

Throughout the full text, we suppose that

1.3 $$\begin{aligned}& \begin{aligned}&p_{3} =\frac{32 ( 30-\pi^{2} ) }{15\pi^{6}}\approx 0.04467, \\ &p_{2} =\frac{4}{70\text{,}875}\approx 5. 643 7 \times 10^{-5}, \\ &p_{1} =\frac{64 ( 1-\pi^{2}/30-e^{-2/5} ) }{\pi^{6}}\approx 4.6143\times 10^{-5}, \\ &p_{0} \approx 3.799533\times 10^{-5}, \end{aligned} \end{aligned}$$

where $p_{0}$ is the unique zero of the function

1.4 $$ h ( p ) =\ln \biggl( 1-\frac{\pi^{2}}{30}-\frac{\pi^{6}}{64}p \biggr) - \frac{8(45\pi^{4}p+32)}{15 \pi^{6}p+32\pi^{2}-960} $$

on $( 0,p_{2} ) $.

Now considering the asymptotic expansion of $( e^{x\cot x-1} ) ^{2/5}$, we have

$$\bigl( e^{x\cot x-1} \bigr) ^{2/5}=1-\frac{2}{15}x^{2}- \frac{4}{70\text{,}875}x^{6}+\frac{2}{1\text{,}063\text{,}125}x^{8}+O \bigl( x^{10} \bigr). $$

It is interesting that the power series above has not item of $x^{4}$, which also remind us to establish a more accurate estimate for $\exp ( x\cot x-1 ) $. The first aim of this paper is to determine the best parameters *p* and *q* such that the double inequality

$$\biggl( 1-\frac{2}{15}x^{2}-px^{6} \biggr) ^{5/2}< e^{x\cot x-1}< \biggl( 1- \frac{2}{15}x^{2}-qx^{6} \biggr) ^{5/2} $$

holds for all $x\in ( 0,\pi /2 ) $. The main conclusions of this paper are proved by the recursive method and a new criterion for the monotonicity of the quotient of two power series. The following result is a theorem on the recurrence relation of coefficients in the series expansion of the function $\ln (1-2x^{2}/15-px^{6})$.

### Theorem 1

*Let* $0< p< p_{3}$ *and* $x\in ( 0,\pi /2 ) $. *Then the function* $f ( x ) =\ln ( 1-2x^{2}/15-px^{6} ) $ *can be expressed in the form of a series*,

1.5 $$ \ln \biggl( 1-\frac{2}{15}x^{2}-px^{6} \biggr) =-\sum _{n=1}^{\infty }a _{n}x^{2n}, $$

*where*

1.6 $$ a_{1}=\frac{2}{15},\qquad a_{2}=\frac{2}{225},\qquad a_{3}=p+ \frac{8}{10\text{,} 125} , $$

*and*, *for* $n\geq 3$,

1.7 $$ a_{n+1}=\frac{2n}{15 ( n+1 ) }a_{n}+p\frac{n-2}{n+1}a_{n-2}. $$

*Moreover*, $a_{n}>0$ *for all* $n\geq 1$.

Our main results are contained in the following theorems.

### Theorem 2

*Let* $0< p< p_{3}$ *and* $x\in ( 0,\pi /2 ) $.

(i) *If* $p_{2}\leq p< p_{3}$, *then the function*
  $$x\mapsto \frac{\ln ( 1-2x^{2}/15-px^{6} ) }{x\cot x-1}:=\frac{f ( x ) }{g ( x ) } $$
  *is strictly increasing on* $( 0,\pi /2 ) $, *and therefore the double inequality*
  1.8 $$ \biggl( 1-\frac{2}{15}x^{2}-px^{6} \biggr) ^{5/2}< e^{x\cot x-1}< \biggl( 1- \frac{2}{15}x^{2}-px^{6} \biggr) ^{1/\lambda_{p}} $$
  *holds*, *where*
  $$\lambda_{p}=-\ln \biggl( 1-\frac{\pi^{2}}{30}-\frac{\pi^{6}}{64}p \biggr). $$
(ii) *If* $p_{0}< p< p_{2}$, *then there is an* $x_{0}\in ( 0,\pi /2 ) $ *such that the function* $f/g$ *is strictly decreasing on* $( 0,x_{0} ) $ *and strictly increasing on* $( x_{0}, \pi /2 ) $. *Consequently*, *the inequality*
  1.9 $$ e^{x\cot x-1}< \biggl( 1-\frac{2}{15}x^{2}-px^{6} \biggr) ^{1/\theta_{p}} $$
  *holds*, *where* $\theta_{p}=\max ( 2/5,\lambda_{p} ) $. *In particular*, *we have*
  1.10 $$\begin{aligned}& e^{x\cot x-1} < \biggl( 1-\frac{2}{15}x^{2}-px^{6} \biggr) ^{5/2} \quad \textit{for }p_{0}< p\leq p_{1}, \end{aligned}$$
  1.11 $$\begin{aligned}& e^{x\cot x-1} < \biggl( 1-\frac{2}{15}x^{2}-px^{6} \biggr) ^{1/\lambda _{p}}\quad \textit{for }p_{1}< p< p_{2}. \end{aligned}$$
(iii) *If* $0< p< p_{0}$, *then the function* $f/g$ *is strictly decreasing on* $( 0,\pi /2 ) $, *and therefore the double inequality* (1.8) *is reversed*.

As a consequence of Theorem 2, we immediately get the following.

### Theorem 3

*Let* $p_{2}\leq p< p_{3}$ *and* $p_{0}< q\leq p_{1}$. *Then the double inequality*

1.12 $$ \biggl( 1-\frac{2}{15}x^{2}-px^{6} \biggr) ^{5/2}< e^{x\cot x-1}< \biggl( 1- \frac{2}{15}x^{2}-qx^{6} \biggr) ^{5/2} $$

*holds for all* $x\in (0,\pi /2)$ *with the best coefficients* $p=p_{2}$ *and* $q=p_{1}$. *In particular*, *we have*

$$\biggl( 1-\frac{2}{15}x^{2}-\frac{4}{70\text{,}875}x^{6} \biggr) ^{5/2}< e^{x \cot x-1}< \biggl( 1-\frac{2}{15}x^{2}- \frac{64 ( 1-\pi^{2}/30-e ^{-2/5} ) }{\pi^{6}}x^{6} \biggr) ^{5/2} $$

*for all* $x\in (0,\pi /2)$.

The second aim of this paper is to refine some known results presented in [19], we shall state it carefully in the fifth section.

## Lemmas

In this paper, we will use some methods, such as the monotone form of l’Hospital’s rule, an important criterion for the monotonicity of the quotient of two power series, and the latest promotion of the latter.

### Lemma 1

([20, 21])

*For* $-\infty < a< b<\infty $, *let* $f,g:[a,b]\rightarrow \mathbb{R}$ *be continuous functions that are differentiable on* $( a,b ) $, *with* $f ( a ) =g ( a ) =0$ *or* $f ( b ) =g ( b ) =0$. *Assume that* $g^{\prime }(x)\neq 0$ *for each* *x* *in* $(a,b)$. *If* $f^{\prime }/g^{\prime }$ *is increasing* (*decreasing*) *on* $(a,b)$, *then so is* $f/g$.

### Lemma 2

([22–24])

*Let* $A ( t ) =\sum_{k=0}^{\infty }a_{k}t^{k}$ *and* $B ( t ) =\sum_{k=0}^{\infty }b_{k}t^{k}$ *be two real power series converging on* $( -r,r ) $ ($r>0$) *with* $b_{k}>0$ *for all* *k*. *If the sequence* $\{a_{k}/b_{k}\} $ *is increasing* (*decreasing*) *for all* *k*, *then the function* $t\mapsto A ( t ) /B ( t ) $ *is also increasing* (*decreasing*) *on* $( 0,r ) $.

Now, we will introduce a useful auxiliary function $H_{f,g}$. For $-\infty \leq a< b\leq \infty $, let *f* and *g* be differentiable on $(a,b)$ and $g^{\prime }\neq 0$ on $(a,b)$. Then the function $H_{f,g}$ is defined by

2.1 $$ H_{f,g}:=\frac{f^{\prime }}{g^{\prime }}g-f. $$

The function $H_{f,g}$ has some good properties [25, Property 1] and plays an important role in the proof of a monotonicity criterion for the quotient of power series (see [26]).

### Lemma 3

([26, Theorem 1])

*Let* $A ( t ) =\sum_{k=0}^{\infty }a_{k}t^{k}$ *and* $B ( t ) =\sum_{k=0}^{\infty }b_{k}t^{k}$ *be two real power series converging on* $( -r,r ) $ *and* $b_{k}>0$ *for all* *k*. *Suppose that for certain* $m\in \mathbb{N} $, *the non*-*constant sequence* $\{ a_{k}/b_{k} \} $ *is increasing* (*resp*. *decreasing*) *for* $0\leq k\leq m$ *and decreasing* (*resp*. *increasing*) *for* $k\geq m$. *Then the function* $A/B$ *is strictly increasing* (*resp*. *decreasing*) *on* $( 0,r ) $ *if and only if* $H_{A,B} ( r^{-} ) \geq $ (*resp*. ≤) 0. *Moreover*, *if* $H_{A,B} ( r^{-} ) <$ (*resp*. >) 0, *then there exists* $t_{0}\in ( 0,r ) $ *such that the function* $A/B$ *is strictly increasing* (*resp*. *decreasing*) *on* $( 0,t_{0} ) $ *and strictly decreasing* (*resp*. *increasing*) *on* $( t_{0},r ) $.

### Lemma 4

([27])

*For* $n\in N$, *the Bernoulli numbers satisfy*

2.2 $$ \frac{1}{ ( 2\pi ) ^{2}}\frac{2n ( 2n-1 ) ( 2^{2n-3}-1 ) }{2^{2n-3}}< \frac{\vert B_{2n}\vert }{ \vert B_{2n-2}\vert }< \frac{1}{ ( 2\pi ) ^{2}} \frac{2n ( 2n-1 ) 2^{2n-1}}{2^{2n-1}-1}. $$

### Lemma 5

*For* $0< p< p_{2}=4/70\text{,}875$, *let* $h(p)$ *be defined by* (1.4). *Then* $h ( p ) $ *has a unique zero* $p_{0}\approx 3.799533\times 10^{-5}$ *such that* $h ( p ) <0$ *for* $p\in ( 0,p_{0} ) $ *and* $h ( p ) >0$ *for* $p\in ( p_{0},p_{2} ) $.

### Proof

Differentiation yields

$$h^{\prime } ( p ) =15\pi^{4}\frac{15\pi^{8}p+32\pi^{4}-1472 \pi^{2}+23\text{,}040}{ ( 15\pi^{6}p+32\pi^{2}-960 ) ^{2}}>0, $$

which together with the facts that

$$\begin{aligned} h \bigl( 0^{+} \bigr) =&\ln \biggl( 1-\frac{\pi^{2}}{30} \biggr) - \frac{8}{ \pi^{2}-30}\approx -0.001557 5< 0, \\ h ( p_{2} ) =&h \biggl( \frac{4}{70\text{,}875} \biggr) =\ln \biggl( 1- \frac{ \pi^{2}}{30}-\frac{\pi^{6}}{11\text{,}340\text{,}000} \biggr) -\frac{24\pi^{4}+3\text{,}024\text{,} 000}{378\text{,}000\pi^{2}+\pi^{6}-11\text{,}340\text{,}000} \\ \approx &0.0007572>0, \end{aligned}$$

reveals that there is a unique $p_{0}\in ( 0,p_{2} ) $ such that $h ( p ) <0$ for $p\in ( 0,p_{0} ) $ and $h ( p ) >0$ for $p\in ( p_{0},p_{2} ) $. Numerically, the equation $h ( p ) =0$ for *p* on $( 0,p_{2} ) $ has the solution $p_{0}\approx 3.799533\times 10^{-5}$. This completes the proof. □

## Proof of Theorem 1

### Proof

Since $0< p< p_{3}$ and $x\in ( 0,\pi /2 ) $, we see that

$$0< \frac{2}{15}x^{2}+px^{6}< 1, $$

which shows that

3.1 $$ \ln \biggl( 1-\frac{2}{15}x^{2}-px^{6} \biggr) =-\sum _{n=1}^{\infty } \frac{1}{n} \biggl( \frac{2}{15}x^{2}+px^{6} \biggr) ^{n}:=-\sum _{n=1} ^{\infty }a_{n}x^{2n} $$

holds for all $x\in ( 0,\pi /2 ) $. It remains to determine the coefficients $a_{n}$. Differentiation for the two sides of (3.1) gives

$$\frac{90px^{5}+4x}{15px^{6}+2x^{2}-15}=-\sum_{n=1}^{\infty }2na_{n}x ^{2n-1}, $$

which is equivalent to

$$\begin{aligned} 45px^{4}+2 =&- \bigl( 15px^{6}+2x^{2}-15 \bigr) \sum_{n=1}^{\infty }na _{n}x^{2n-2} \\ =&15a_{1}+ ( 30a_{2}-2a_{1} ) x^{2}+ ( 45a_{3}-4a_{2} ) x^{4} \\ &{}+\sum_{n=3}^{\infty } \bigl[ 15 ( n+1 ) a_{n+1}-15p ( n-1 ) a_{n-2}-2na_{n} \bigr] x^{2n}. \end{aligned}$$

Comparing coefficients gives the recurrence formulas (1.7) and (1.6).

From the second equality of (3.1) we easily find that $a_{n}>0$ for all $n\geq 1$, which completes the proof. □

## Proofs of Theorems 2 and 3

### Proof of Theorem 2

Using the expansion

$$g ( x ) =x\cot x-1=-\sum_{n=1}^{\infty } \frac{2^{2n}}{ ( 2n )!}\vert B_{2n}\vert x^{2n},\quad \vert x \vert < \pi, $$

the function $f/g$ can be expressed as

$$\frac{f ( x ) }{g ( x ) }=\frac{\ln ( 1-2x^{2}/15-px ^{6} ) }{x\cot x-1}=\frac{-\sum_{n=1}^{\infty }a_{n}x^{2n}}{- \sum_{n=1}^{\infty }\frac{2^{2n}}{ ( 2n )!}\vert B_{2n}\vert x ^{2n}}:=\frac{\sum_{n=1}^{\infty }a_{n}t^{n}}{\sum_{n=1}^{\infty }b _{n}t^{n}} $$

by Theorem 1, where $x^{2}=t$. We now observe the monotonicity of the sequence $\{a_{n}/b_{n}\}_{n\geq 1}$. Since $b_{n}>0$ for all $n\geq 1$, it suffices to determine the sign of $c_{n}:=a_{n+1}- ( b_{n+1}/b_{n} ) a_{n}$. Direct computations yield

$$\begin{aligned}& c_{1} =a_{2}-\frac{b_{2}}{b_{1}}a_{1}=0, \\ & c_{2} =a_{3}-\frac{b_{3}}{b_{2}}a_{2}=p- \frac{4}{70\text{,}875}. \end{aligned}$$

We claim that $c_{n}>0$ for $n\geq 3$. In fact, by means of the recurrence formula (1.7), we have

$$\begin{aligned} c_{n} =&a_{n+1}-\frac{b_{n+1}}{b_{n}}a_{n} \\ =&a_{n+1}- \frac{2}{ ( n+1 ) ( 2n+1 ) }\frac{\vert B_{2n+2}\vert }{ \vert B_{2n}\vert }a_{n} \\ =&\frac{2}{n+1} \biggl( \frac{n}{15}-\frac{1}{2n+1} \frac{\vert B _{2n+2}\vert }{\vert B_{2n}\vert } \biggr) a_{n}+p \frac{n-2}{n+1}a_{n-2}. \end{aligned}$$

Clearly, if we prove

$$d_{n}=\frac{n}{15}-\frac{1}{2n+1}\frac{\vert B_{2n+2}\vert }{ \vert B_{2n}\vert }>0 $$

for $n\geq 3$, then it follows that $c_{n}>0$. Using the right hand side of (2.2) we have

$$\begin{aligned} d_{n} >&\frac{n}{15}-\frac{1}{2n+1} \frac{1}{ ( 2\pi ) ^{2}} \frac{2 ( n+1 ) ( 2n+1 ) 2^{2n+1}}{2^{2n+1}-1} \\ >&\frac{n}{15}-\frac{1}{2n+1}\frac{ ( n+1 ) ( 2n+1 ) }{4\times 19/2}\frac{2^{2n}-1}{2^{2n+1}-1} \\ =&\frac{1}{285}\frac{ ( 8n-30 ) 2^{2n}-19n}{2^{2n+1}-1}>0 \end{aligned}$$

for $n\geq 4$. This together with $d_{3}=0$ yields $d_{n}\geq 0$ for $n\geq 3 $.

(i) If $p_{2}\leq p< p_{3}$, then $c_{n}=a_{n+1}- ( b_{n+1}/b_{n} ) a_{n}>0$ for $n\geq 1$, that is, the sequence $\{a_{n}/b_{n}\}_{n\geq 1}$ is strictly increasing, so is $f/g$ on $( 0,\pi /2 ) $ by Lemma 2. Therefore, we conclude that
  $$\frac{2}{5}=\lim_{x\rightarrow 0^{+}}\frac{f ( x ) }{g ( x ) }< \frac{f ( x ) }{g ( x ) }< \lim_{x\rightarrow ( \pi /2 ) ^{-}}\frac{f ( x ) }{g ( x ) }=-\ln \biggl( 1- \frac{\pi^{2}}{30}-\frac{\pi^{6}}{64}p \biggr) =\lambda_{p}, $$
  which implies (1.8).
(ii) If $0< p< p_{2}$, then $c_{1}=0$, $c_{2}<0$ and $c_{n}>0$ for $n\geq 3$, which indicates that the sequence $\{a_{n}/b_{n}\}$ is decreasing for $n=1,2,3$ and increasing for $n\geq 3$. By Lemma 3, to determine the monotonicity of $f/g$ on $( 0, \pi /2 ) $, we have to observe the sign of $H_{-f ( \sqrt{t} ),-g ( \sqrt{t} ) } ( ( \pi^{2}/4 ) ^{-} ) $. A simple computation leads us to
  $$\begin{aligned} H_{-f ( \sqrt{t} ),-g ( \sqrt{t} ) } \biggl( \biggl( \frac{\pi^{2}}{4} \biggr) ^{-} \biggr) =& \lim_{t\rightarrow ( \pi^{2}/4 ) ^{-}} \biggl[ \frac{-f^{ \prime } ( \sqrt{t} ) }{-g^{\prime } ( \sqrt{t} ) } \bigl( -g ( \sqrt{t} ) \bigr) +f ( \sqrt{t} ) \biggr] \\ =&\ln \biggl( 1-\frac{\pi^{2}}{30}-\frac{\pi^{6}}{64}p \biggr) - \frac{8(45 \pi^{4}p+32)}{15\pi^{6}p+32\pi^{2}-960} \\ =&h ( p ). \end{aligned}$$

*Subcase 2.1*: For $p_{0}< p< p_{1}$. By Lemma 5 we see that $H_{-f ( \sqrt{t} ),-g ( \sqrt{t} ) } ( ( \pi^{2}/4 ) ^{-} ) >0$. It follows from Lemma 3 that there is an $t_{0}\in ( 0,\pi^{2}/4 ) $ such that the function $-f ( \sqrt{t} ) / ( -g ( \sqrt{t} ) ) $ is strictly decreasing on $( 0,t_{0} ) $ and strictly increasing on $( t_{0},\pi /2 ) $, where $t=x^{2}$. Consequently, we obtain

$$\frac{f ( x ) }{g ( x ) }< \max \biggl( \lim_{x\rightarrow 0^{+}}\frac{f ( x ) }{g ( x ) }, \lim_{x\rightarrow ( \pi /2 ) ^{-}}\frac{f ( x ) }{g ( x ) } \biggr) =\max \biggl( \frac{2}{5},\lambda_{p} \biggr), $$

which implies (1.9).

In particular, if $\lambda_{p}\leq 2/5$, that is, $p\in (0,p_{0}]$, then the inequality (1.10) holds. If $\lambda_{p}>2/5$, that is, $p\in ( p_{0},p_{1} ) $, then the inequality (1.11) holds.

*Subcase 2.2*: For $0< p\leq p_{0}$. By Lemma 5 we see that $H_{-f ( \sqrt{t} ),-g ( \sqrt{t} ) } ( ( \pi^{2}/4 ) ^{-} ) \leq 0$. From Lemma 3 it is deduced that $f/g$ is strictly decreasing on $( 0,\pi /2 ) $, and so the inequalities (1.8) reverse. This completes the proof. □

### Proof of Theorems 3

Let

$$H(p,x)= \biggl( 1-\frac{2}{15}x^{2}-px^{6} \biggr) ^{5/2},\quad 0< x< \frac{ \pi }{2},0< p< p_{3}. $$

Since

$$\frac{\partial }{\partial p}H(p,x)= -\frac{5}{2}x^{6} \biggl( 1-\frac{2}{15}x^{2}-px^{6} \biggr) ^{3/2}< 0, $$

we find that the function $H(p,x)$ is decreasing with respect to *p* on $(0,p_{3})$. Then by the left hand side of (1.8) and by (1.10) we can complete the proof of Theorem 3. □

## Consequences and remarks

### Remark 1

One can obtain the double inequality (1.12) using the key theorem of Wu and Debnath [28].

Let $p\rightarrow 0^{+}$ in (iii) of Theorem 2. Then we have the following.

### Corollary 1

*The function*

$$x\mapsto F_{2/15} ( x ) =\frac{\ln ( 1-2x^{2}/15 ) }{x\cot x-1} $$

*is strictly decreasing on* $( 0,\pi /2 ) $, *and therefore*, *the inequalities*

5.1 $$ \alpha \biggl( 1-\frac{2}{15}x^{2} \biggr) ^{5/2}< \biggl( 1-\frac{2}{15}x ^{2} \biggr) ^{1/\lambda_{0}}< \exp ( x\cot x-1 ) < \biggl( 1- \frac{2}{15}x^{2} \biggr) ^{5/2} $$

*hold for* $x\in ( 0,\pi /2 ) $, *where*

$$\begin{aligned} \lambda_{0} =&-\ln \biggl( 1-\frac{\pi^{2}}{30} \biggr) \approx 0.39897, \\ \alpha =&e^{-1} \bigl( 1-\pi^{2}/30 \bigr) ^{-5/2} \approx 0.997 42, \end{aligned}$$

*are the best constants*.

### Proof

It suffices to show the first inequality in (5.1). Consider the monotonicity of the function

$$K(x)= \biggl( 1-\frac{2}{15}x^{2} \biggr) ^{1/\lambda_{0}-5/2} $$

on $( 0,\pi /2 ) $. Since

$$K^{\prime }(x)= \biggl( \frac{1}{\lambda_{0}}-\frac{5}{2} \biggr) \biggl( -\frac{4}{15}x \biggr) \biggl( 1-\frac{2}{15}x^{2} \biggr) ^{1/\lambda_{0}-7/2}< 0 $$

holds for all $x\in ( 0,\pi /2 ) $, we see that the function $K(x)$ is decreasing on $( 0,\pi /2 ) $. So

$$K(x)>K\bigl( ( \pi /2 ) ^{-}\bigr)=e^{-1} \bigl( 1- \pi^{2}/30 \bigr) ^{-5/2}=\alpha, $$

which completes the proof of Corollary 1. □

### Theorem 4

*Let* $0< p\leq 4/\pi^{2}$. *Then the function*

5.2 $$ x\mapsto F_{p} ( x ) =\frac{\ln ( 1-px^{2} ) }{x \cot x-1} $$

*is strictly decreasing on* $( 0,\pi /2 ) $ *if and only if* $0< p\leq 2/15$. *And therefore*, *for* $0< p\leq 2/15$, *the double inequality*

5.3 $$ \bigl( 1-px^{2} \bigr) ^{1/\beta_{p}}< e^{x\cot x-1}< \bigl( 1-px^{2} \bigr) ^{1/ ( 3p ) } $$

*holds for* $x\in ( 0,\pi /2 ) $, *where* $\beta_{p}=-\ln ( 1-p\pi^{2}/4 ) $.

### Proof

The necessity follows from

$$\lim_{x\rightarrow 0+}\frac{F_{p}^{\prime } ( x ) }{x}= \frac{1}{5}p ( 15p-2 ) \leq 0. $$

To prove the sufficiency, we note that

$$\frac{\ln ( 1-px^{2} ) }{x\cot x-1}=\frac{\ln ( 1-2x ^{2}/15 ) }{x\cot x-1}\times \frac{\ln ( 1-px^{2} ) }{ \ln ( 1-2x^{2}/15 ) }:=f_{1} ( x ) \times f_{2} ( x ), $$

where $f_{1}$ is positive and decreasing on $( 0,\pi /2 ) $ by Corollary 1, it thus suffices to prove the function $f_{2}$ is positive and decreasing on $( 0,\pi /2 ) $. A simple computation gives

$$\begin{aligned}& \frac{ [ \ln ( 1-px^{2} ) ] ^{\prime }}{ [ \ln ( 1-2x^{2}/15 ) ] ^{\prime }} =\frac{1}{2}p\frac{15-2x ^{2}}{1-px^{2}}, \\& \biggl( \frac{1}{2}p\frac{15-2x^{2}}{1-px^{2}} \biggr) ^{\prime } =15px \frac{p-2/15}{ ( px^{2}-1 ) ^{2}}< 0, \end{aligned}$$

which indicates that $f_{2}$ is strictly decreasing on $( 0, \pi /2 ) $ by Lemma 1. Meanwhile $f_{2} ( x ) $ is obviously positive for $p\in (0,2/15]$, which proves the sufficiency.

Inequalities (5.3) follow from the decreasing property of the function $F_{p} ( x ) $.

The proof is finished. □

### Remark 2

It is easy to check that, for $0< p\leq 2/15$ and $x\in ( 0, \pi /2 ) $,

5.4 $$ \bigl( 1-px^{2} \bigr) ^{1/\beta_{p}}>\alpha_{p} \bigl( 1-px^{2} \bigr) ^{1/ ( 3p ) }, $$

where $\alpha_{p}=e^{-1} ( 1-\pi^{2}p/4 ) ^{-1/ ( 3p ) }$, $\beta_{p}=-\ln ( 1-p\pi^{2}/4 ) $. In fact, we have

$$\begin{aligned}& \frac{\ln ( 1-px^{2} ) }{\beta_{p}}-\ln \alpha_{p}- \frac{1}{3p}\ln \bigl( 1-px^{2} \bigr) \\& \quad =\frac{\ln ( 1-px^{2} ) }{-\ln ( 1-p\pi^{2}/4 ) }+1+\frac{1}{3p}\ln \bigl( 1-p\pi^{2}/4 \bigr) -\frac{1}{3p}\ln \bigl( 1-px^{2} \bigr) \\& \quad =\frac{1}{\ln ( 1-p\pi^{2}/4 ) }\ln \frac{1-p\pi^{2}/4}{1-px ^{2}}+\frac{1}{3p}\ln \frac{1-p\pi^{2}/4}{1-px^{2}} \\& \quad = \biggl[ 1-\frac{\ln ( 1-px^{2} ) }{\ln ( 1-p\pi^{2}/4 ) } \biggr] \biggl[ 1+\frac{\ln ( 1-p\pi^{2}/4 ) }{3p} \biggr] . \end{aligned}$$

Due to $x\in ( 0,\pi /2 ) $, the first factor is positive. And, since $p\mapsto 1+ ( \ln ( 1-p\pi^{2}/4 ) ) / ( 3p ) $ is decreasing in *p*, it follows that, for $0< p\leq 2/15$,

$$\biggl( 1+\frac{\ln ( 1-p\pi^{2}/4 ) }{3p} \biggr) > \frac{5}{2}\ln \biggl( 1- \frac{\pi^{2}}{30} \biggr) +1\approx 0.002583 8>0. $$

These imply that the inequality (5.4) holds. It thus can be seen that the above theorem partly refines Lv et al.’s result [19].

### Remark 3

We claim that the lower bound in the double inequality (5.3) is strictly increasing with respect to the parameter *p*. In fact, put

$$\ln \bigl( 1-px^{2} \bigr) ^{1/\beta_{p}}=\frac{\ln ( 1-px^{2} ) }{-\ln ( 1-p\pi^{2}/4 ) }:= \frac{h_{1} ( p ) }{h_{2} ( p ) } $$

with $h_{1} ( 0^{+} ) =h_{2} ( 0^{+} ) =0$, then differentiation yields

$$\begin{aligned}& \frac{h_{1}^{\prime } ( p ) }{h_{2}^{\prime } ( p ) } =\frac{\frac{d}{dp}\ln ( 1-px^{2} ) }{-\frac{d}{dp} \ln ( 1-p\pi^{2}/4 ) }=-\frac{x^{2}}{\pi^{2}}\frac{4-\pi ^{2}p}{1-px^{2}}, \\& \biggl( \frac{h_{1}^{\prime } ( p ) }{h_{2}^{\prime } ( p ) } \biggr) ^{\prime } =\frac{x^{2}}{\pi^{2}} \frac{\pi^{2}-4x ^{2}}{ ( 1-px^{2} ) ^{2}}>0, \end{aligned}$$

which indicates that $h_{1}/h_{2}$ is increasing in *p* by Lemma 1.

### Remark 4

Taking $p=0^{+}$, $1/\pi^{2}$, and $2/15$ in Theorem 4. Using the monotonicity of the lower and upper bounds in (5.3), we can obtain

$$\begin{aligned} e^{-4x^{2}/\pi^{2}} < & \biggl( 1-\frac{x^{2}}{\pi^{2}} \biggr) ^{1/ \ln ( 4/3 ) }< \biggl( 1-\frac{2}{15}x^{2} \biggr) ^{1/\beta_{2/15}}< e ^{x\cot x-1} \\ < & \biggl( 1-\frac{2}{15}x^{2} \biggr) ^{5/2}< \biggl( 1-\frac{x^{2}}{\pi ^{2}} \biggr) ^{\pi^{2}/3}< e^{-x^{2}/3}, \end{aligned}$$

where $\beta_{2/15}=-\ln ( 1-\pi^{2}/30 ) \approx 0.398 97$. This shows that our double inequality (5.3) is a generalization and refinement of the one (1.2) (see [19]).

The following theorem gives a sufficient condition for the function $F_{p} ( x ) $ to be increasing on $( 0,\pi /2 ) $.

### Theorem 5

*The function* $F_{p} ( x ) $ *defined by* (5.2) *is strictly increasing on* $( 0,\pi /2 ) $ *if* $1/7\leq p\leq 4/\pi^{2}$. *And therefore*, *for* $1/7\leq p\leq 4/\pi^{2}$, *the double inequality* (5.3) *is reversed*.

### Proof

We have

$$\begin{aligned}& F_{p} ( x ) =\frac{-\ln ( 1-px^{2} ) }{- ( x \cot x-1 ) }=\frac{\sum_{n=1}^{\infty }\frac{p^{n}}{n}x^{2n}}{ \sum_{n=1}^{\infty }\frac{2^{2n}}{ ( 2n )!}\vert B_{2n}\vert x ^{2n}}:= \frac{\sum_{n=1}^{\infty }a_{n}^{\prime }x^{2n}}{\sum_{n=1} ^{\infty }b_{n}^{\prime }x^{2n}}, \\& c_{n}^{\prime } =a_{n+1}^{\prime }- \frac{b_{n+1}^{\prime }}{b_{n} ^{\prime }}a_{n}^{\prime } \\& \hphantom{c_{n}^{\prime }}=\frac{p^{n+1}}{n+1}- \frac{2}{ ( n+1 ) ( 2n+1 ) }\frac{\vert B_{2n+2}\vert }{ \vert B_{2n}\vert }\frac{p^{n}}{n} \\& \hphantom{c_{n}^{\prime }} =\frac{p^{n}}{n+1} \biggl( p-\frac{2}{n ( 2n+1 ) }\frac{ \vert B_{2n+2}\vert }{\vert B_{2n}\vert } \biggr):=\frac{p^{n}}{n+1} ( p-u_{n} ). \end{aligned}$$

If we prove $( p-u_{n} ) \geq 0$ for $n\geq 1$, then by Lemma 2 $F_{p}$ is increasing on $( 0,\pi /2 ) $, and the reverse of double inequality (5.3) follows. Using the right hand side of (2.2) we have

$$\begin{aligned} p-u_{n} \geq &\frac{1}{7}-\frac{2}{n ( 2n+1 ) } \frac{1}{ ( 2\pi ) ^{2}} \frac{2 ( n+1 ) ( 2n+1 ) 2^{2n+1}}{2^{2n+1}-1} \\ =&\frac{1}{7\pi^{2}}\frac{ ( ( \pi^{2}-7 ) n-7 ) 2^{2n+1}- \pi^{2}n}{n ( 2^{2n+1}-1 ) }>0 \end{aligned}$$

for $n\geq 3$. This together with $p-u_{1}=p-2/15>0$ and $p-u_{2}=p-1/7 \geq 0 $ yields $( p-u_{n} ) \geq 0$ for $n\geq 1$.

This completes the proof. □

### Remark 5

Likewise, taking $p=1/7$, $1/3$, $4/\pi^{2}$ in the above theorem, we have

$$\begin{aligned} \biggl( 1-\frac{4x^{2}}{\pi^{2}} \biggr) ^{\pi^{2}/12} < &1- \frac{x^{2}}{3}< \biggl( 1-\frac{x^{2}}{7} \biggr) ^{7/3}< e^{x\cot x-1} \\ < & \biggl( 1-\frac{x^{2}}{7} \biggr) ^{1/\beta_{1/7}}< \biggl( 1- \frac{x ^{2}}{3} \biggr) ^{1/\beta_{1/3}}, \end{aligned}$$

where $\beta_{1/7}=-\ln ( 1-\pi^{2}/28 ) \approx 0.434 61$, $\beta_{1/3}=-\ln ( 1-\pi^{2}/12 ) \approx 1.728 6$.

Finally, we consider the monotonicity of the function $F_{p} ( x ) $ on $( 0,\pi ) $. In this case, we have to assume that $0< p\leq 1/\pi^{2}$.

### Theorem 6

*Let* $0< p\leq 1/\pi^{2}$. *Then the function* $F_{p} ( x ) $ *defined by* (5.2) *is strictly decreasing on* $( 0,\pi ) $. *And therefore*, *the inequality*

$$e^{x\cot x-1}< \bigl( 1-px^{2} \bigr) ^{1/ ( 3p ) } $$

*holds for all* $x\in ( 0,\pi ) $.

### Proof

From Lemma 2 and the proof of the previous theorem, it suffices to prove $p-u_{n}\leq 0$ for $n\geq 1$ and $0< p\leq 1/\pi ^{2}$. Using the left hand side of (2.2) we get

$$\begin{aligned} p-u_{n} =&p-\frac{2}{n ( 2n+1 ) }\frac{\vert B_{2n+2}\vert }{ \vert B_{2n}\vert } \\ \leq &\frac{1}{\pi^{2}}-\frac{2}{n ( 2n+1 ) }\frac{1}{ ( 2\pi ) ^{2}} \frac{2 ( n+1 ) ( 2n+1 ) ( 2^{2n-1}-1 ) }{2^{2n-1}} \\ =&-\frac{2^{2n}-2n-2}{2^{2n}\pi^{2}n}\leq 0 \end{aligned}$$

for $n\geq 1$, which, by Lemma 2, proves the decreasing property of $F_{p}$. □

### Remark 6

Let $p=p_{1}=32 ( 30-\pi^{2}-30e^{-2/5} ) / ( 15\pi^{6} ) \approx 4.614 3\times 10^{-5}$ in Theorem 3, we can obtain

$$e^{x\cot x-1}< \biggl( 1-\frac{2}{15}x^{2}-p_{1}x^{6} \biggr) ^{5/2},\quad 0< x< \frac{ \pi }{2}, $$

which is sharper than the right hand side of (1.2):

$$e^{x\cot x-1}< \biggl( 1-\frac{2}{15}x^{2} \biggr) ^{5/2},\quad 0< x< \frac{ \pi }{2}. $$

### Remark 7

Let $p=p_{2}=4/70\text{,}875$ in Theorem 2 or Theorem 3, we can obtain

$$\biggl( 1-\frac{2}{15}x^{2}-\frac{4}{70\text{,}875}x^{6} \biggr) ^{5/2}< e^{x \cot x-1},\quad 0< x< \frac{\pi }{2}. $$

Comparing the inequality above with the left hand side of (1.2), we find that they are not included in each other.

## Conclusions

In the present study, we first obtain some new bounds for the exponential function with cotangent by using the recurrence relation between coefficients in the expansion of power series of the function $\ln (1-2x^{2}/15-px^{6})$ and a new criterion for the monotonicity of the quotient of two power series. Then we refine some well-known results presented in [19].
